# Freezing in a warming climate: Marked declines of a subnivean hibernator after a snow drought

**DOI:** 10.1002/ece3.7126

**Published:** 2020-12-29

**Authors:** Aaron N. Johnston, Roger G. Christophersen, Erik A. Beever, Jason I. Ransom

**Affiliations:** ^1^ U. S. Geological Survey Northern Rocky Mountain Science Center Bozeman MT USA; ^2^ School of Environmental and Forest Sciences University of Washington Seattle WA USA; ^3^ National Park Service Sedro Woolley WA USA; ^4^ Department of Ecology Montana State University Bozeman MT USA

**Keywords:** American pika, hoary marmot, *Marmota caligata*, *Ochotona princeps*, snow drought, vapor pressure deficit

## Abstract

Recent snow droughts associated with unusually warm winters are predicted to increase in frequency and affect species dependent upon snowpack for winter survival. Changes in populations of some cold‐adapted species have been attributed to heat stress or indirect effects on habitat from unusually warm summers, but little is known about the importance of winter weather to population dynamics and how responses to snow drought vary among sympatric species. We evaluated changes in abundance of hoary marmots (*Marmota caligata*) over a period that included a year of record‐low snowpack to identify mechanisms associated with weather and snowpack. To consider interspecies comparisons, our analysis used the same a priori model set as a concurrent study that evaluated responses of American pikas (*Ochotona princeps*) to weather and snowpack in the same study area of North Cascades National Park, Washington, USA. We hypothesized that marmot abundance reflected mechanisms related to heat stress, cold stress, cold exposure without an insulating snowpack, snowpack duration, atmospheric moisture, growing‐season precipitation, or select combinations of these mechanisms. Changes in marmot abundances included a 74% decline from 2007 to 2016 and were best explained by an interaction of chronic dryness with exposure to acute cold without snowpack in winter. Physiological stress during hibernation from exposure to cold, dry air appeared to be the most likely mechanism of change in marmot abundance. Alternative mechanisms associated with changes to winter weather, including early emergence from hibernation or altered vegetation dynamics, had less support. A post hoc assessment of vegetative phenology and productivity did not support vegetation dynamics as a primary driver of marmot abundance across years. Although marmot and pika abundances were explained by strikingly similar models over periods of many years, details of the mechanisms involved likely differ between species because pika abundances increased in areas where marmots declined. Such differences may lead to diverging geographic distributions of these species as global change continues.

## Graphical abstract

Recent snow droughts associated with unusually warm winters are predicted to increase in frequency and affect species dependent upon snowpack for winter survival. We evaluated changes in abundance of hoary marmots (*Marmota caligata*) over a period that included a year of record‐low snowpack, to identify mechanisms associated with weather and snowpack. Physiological stress during hibernation from exposure to cold, dry air appeared to be the most likely mechanism of change because alternative mechanisms associated with early emergence from hibernation or altered vegetation dynamics had less support.

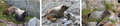

## 1 INTRODUCTION

The worldwide decline in the cryosphere over the past century (IPCC, 2014) has altered hydrological processes that regulate dynamics of mountain and polar ecosystems (Barnett et al., 2005; Dong & Menzel, 2020). Snowpack has declined across mountain ecosystems on many continents (Klein et al., 2016; Mote et al., 2018; Pederson et al., 2013; Vuille et al., 2018) and is predicted to decline further with increasing global temperatures over the next century (Fyfe et al., 2017; Marty et al., 2017; Mote & Salathé, 2010). Reductions in snowpack by 80% relative to 20th‐century averages have been predicted in some regions of North America (Gergel et al., 2017); similar declines have been demonstrated and predicted for nearly every other major mountain chain, globally. Unusually warm weather that causes winter precipitation to fall as rain, rather than snow, recently led to extensive snow droughts in North America (Mote et al., 2016); rain‐on‐snow events are also increasing in frequency throughout much of the globe (Ohba & Kawase, 2020). These unusual conditions are predicted to become normal within this century (Marlier et al., 2017) and lead to profound changes in montane ecosystems.

Winter weather constrains the geographic distribution of many species and has led to adaptations like hibernation or migration (Williams et al., 2015). Snowpack and timing of melt‐off influence water availability, phenology, animal movement, and availability of cover for subnivean biotas that require protective insulation from cold exposure in winter (Pauli et al., 2013; Penczykowski et al., 2017). Ecosystems dominated by snow cover may change rapidly with contemporary climate change because of the strong, widespread effects of small increases in temperature on snowpack, especially in seasons and elevations near the 0°C isotherm (Pepin & Lundquist, 2008). Snow‐removal experiments have revealed significant changes to soil conditions (Groffman et al., 2001), arthropod populations (Templer et al., 2012), arctic lemmings (*Lemmus* and *Dicrostonyx* spp., Reid et al., 2012), and vegetation (Sherwood et al., 2017) to suggest that rapid change in biota can occur following snow drought. Snow droughts could strongly affect a wide variety of snow‐adapted species, including marmots (*Marmota* spp., Armitage, 2013), pikas (*Ochotona* spp., Johnston et al., 2019), wolverines (*Gulo gulo*, Brodie & Post, 2010), wood frogs (*Rana sylvatica*, Sinclair et al., 2013), and Rocky Mountain Apollo butterflies (*Parnassius smintheus*, Roland & Matter, 2013).

Effects of climate change are expected to vary across species based on their sensitivity, exposure, and adaptive capacity to environmental change (Nicotra et al., 2015; Summers et al., 2012). For example, a species’ adaptive capacity reflects attributes related to its life history (e.g., parity, mating system, sex ratio, fecundity), demography, distribution, movement (e.g., site fidelity, migration distance), evolutionary potential, ecological role (e.g., competitive ability, diet breadth), and abiotic niche (Thurman et al., 2020). Winter adaptations like subnivean hibernation can predispose some species to stresses from snow drought and lead to variable responses across species. In addition, within‐species responses to snow drought or other weather events can be context‐dependent across space or time. For example, relationships of American pikas (*Ochotona princeps*) with climate or weather vary across their geographic range (Jeffress et al., 2013; Smith et al., 2019) and within mountain ranges (Beever et al., 2011, 2016; Moyer‐Horner et al., 2016). In‐depth investigations are needed to identify mechanistic relationships of species with climate and weather (Cahill et al., 2012; Ockendon et al., 2014) to posit, test, and refine hypotheses and frameworks that guide prediction of species responses to climate change (e.g., life history, metabolic theory).

Ecological models can improve understanding of species‐climate relationships by considering species’ life history and using predictors that represent mechanisms hypothesized to govern population dynamics (Guisan & Thuiller, 2005; Smith et al., 2019; Wisz et al., 2013). Pikas, for example, are sensitive to heat stress (MacArthur & Wang, 1973), and accordingly, predictors that quantify heat exposure have explained changes in pika distributions in many (Beever et al., 2011, 2013; Stewart et al., 2015) but not all regions (Smith et al., 2019). Likewise, hypothesized effects of snow drought, such as cold stress in the absence of insulating snowpack, can be tested with predictors that intersect data on temperature and snowpack (Johnston et al., 2019). The relative importance of mechanisms related to snow drought, heat stress, and other factors can be evaluated as competing hypotheses in an information‐theoretic approach (Burnham & Anderson, 2002).

Notably, the recent availability of vapor pressure deficit (VPD) in gridded climate data (Daly et al., 2015) has facilitated new, useful models to evaluate mechanisms because VPD represents the drying effect of air on biotas, a pervasive and important factor that determines plant productivity (Oren et al., 1999) and physiological function (Riddell & Sears, 2015) for many species. Furthermore, VPD can mediate effects of extreme temperatures experienced by animals (McArthur, 1991) during snow drought or heat waves. Although the use of VPD in ecological studies has been limited, studies of forests (Williams et al., 2013), grasslands (Konings et al., 2017), salamanders (Riddell et al., 2017), ungulates (Harris et al., 2015), and pikas (Johnston et al., 2019) have demonstrated the strong potential of using VPD to explain ecological phenomena.

Marmot (*Marmota*) species can be useful indicators of change in montane ecosystems because they are among the most climate‐sensitive species (Armitage, 2013; McCain, 2019), and populations of some species can be affected by snowpack dynamics. For example, decreases in litter size of alpine marmots (*M. marmota*, Tafani et al., 2013) and overwinter survival of hoary marmots (*M. caligata*, Patil et al., 2013) have been attributed to poor insulation from surface temperatures due to inadequate snowpack during hibernation. Hypothesized mechanisms for marmot sensitivity to snow drought include fatal exposure to extreme cold in the absence of snowpack (Barash, 1973; Patil et al., 2013), stress from premature emergence from hibernation and lack of forage, and altered phenology or productivity of vegetation that affects forage availability (Inouye et al., 2000; Ozgul et al., 2010). Alternatively, increasing physiological heat stress or indirect effects of warm, dry conditions on forage in summer can negatively affect marmot populations (Armitage, 2013). The influence of winter versus summer weather on marmot populations likely varies across species, geographic ranges, and age classes (Cordes et al., 2020).

Life‐history characteristics of marmots, such as body size and hibernation, may elicit responses to climate change (e.g., changes in distribution, abundance, fecundity, or morphology) that differ from other montane species that are considered indicators for climate change in montane ecosystems (McCain & King, 2014). The also‐sensitive (McCain, 2019) American pika, for example, is sympatric with hoary marmots in some regions, but does not hibernate and has a body mass that is <5% of this marmot species. Johnston et al. (2019) indicated that changes in pika abundances following a snow drought in Washington, USA, varied from negative to positive, according to interactive effects of snowpack and VPD on forage. Pika abundances changed following a 1‐year lag after the snow drought, suggestive of changes to fecundity. It is unknown whether sympatric marmots responded similarly to this snow drought.

We evaluated effects of weather and snowpack dynamics on abundances of hoary marmots over a period that included a year with record‐low snowpack (Mote et al., 2016) during the winter of 2014–2015 (referred to as “winter 2015,” hereafter). Our objectives were to (a) quantify change in marmot abundances over time and (b) evaluate hypothesized mechanisms related to weather and snowpack that may have influenced marmot population dynamics. We predicted that marmot abundances would be lower after the snow drought and that decreases would be greatest among young marmots because of disproportionately high vulnerability of juvenile marmots to stressful winters (Cordes et al., 2020; Patil et al., 2013; Rézouki et al., 2016). We compared a priori models that represented mechanisms concerning exposure to seasonal temperature extremes and precipitation that can affect marmot populations, according to life‐history characteristics and previous studies (Armitage, 2013; McCain, 2019; Patil et al., 2013). We predicted that marmot abundances would decrease following exposure to extreme heat during summer or extreme cold during winter, and we predicted that moist air would minimize heat stress (Beever et al., 2016) but exacerbate cold stress, because condensation can reduce insulative properties of fur (McArthur, 1991). We expected that winter weather and conditions associated with snow drought would have greater influence on marmot abundance than summer conditions. We applied the same model set to marmots that Johnston et al. (2019) used to evaluate pika responses to the 2015 snow drought in the same study area and hypotheses about weather that are applicable to cold‐adapted species like marmots, which offers a useful comparison to examine interspecies variation in mechanisms of vertebrate responses to weather and snowpack.

## 2 MATERIALS AND METHODS

### 2.1 Study area

**FIGURE 1:**
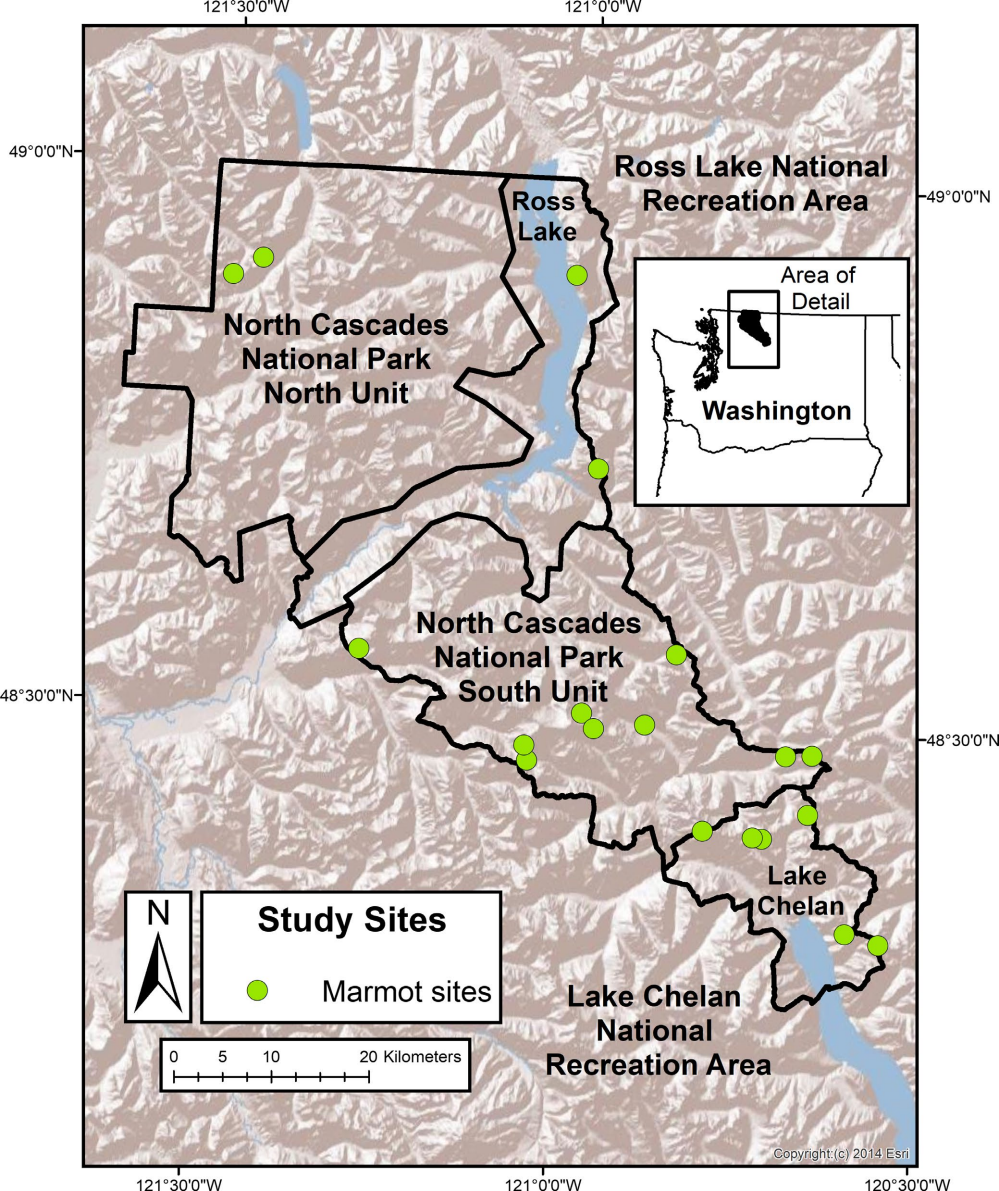
Survey sites for hoary marmots (*Marmota caligata*) in the North Cascades National Park Service Complex, Washington, USA, 2007–2008, 2016, and 2017 (hillshade layer from ESRI, 2014)

The North Cascades National Park Service Complex (hereafter, the Park), which includes North Cascades National Park, Ross Lake National Recreation Area, and Lake Chelan National Recreation Area, is in north‐central Washington, USA (Figure 1). The Park is mostly roadless wilderness with steep mountains, dominated by conifer forests. Elevations range from 100 to 2,800 m. Annual precipitation in this region varies with position relative to mountain divides and ranges from 50 to 500 cm (Mote, 2003). Vegetation associated with wet, dry, or subalpine conditions was juxtaposed because of the topographic complexity. In summers during 2003–2017, mean daily maximum temperatures ranged from 13.7 to 23.9°C and growing‐season precipitation (July–September) ranged from 2.0 to 53.3 cm, across our study sites (Daly et al., 2008). Mean minimum ambient temperatures ranged from −9.7 to −1.5°C in winter, and snow water equivalent ranged from 0 to 93.6 cm on April 1 (Thornton et al., 2017). At the weather station for Thunder Basin (elevation 1,317 m; 48.52 latitude, −120.98 longitude; NRCS, 2017), snow cover was present from 174 to 239 days per winter from 2003–2017, and final melt‐off occurred between 13 May and 27 June. The snow drought of 2015 led to the shortest duration of snowpack (174 days) and earliest date of melt‐off (13 May) during this 15‐year period. Snowpack duration in winter 2004 was the second‐shortest at 196 days; melt‐off occurred that year on 21 May. Hoary marmots generally occupied rocky meadows at elevations >1,200 m in a patchy distribution throughout the Park. American pikas were common in talus and often co‐occurred with marmots.

### 2.2 Field methods

We used point‐transect sampling (Buckland, 2006) to count marmots at 78 stations across 19 sites during each year of the study (Johnston et al., 2020). These sites were among 31 sites that Christophersen (2012) surveyed during 2007 and 2008 to provide baseline information on marmot abundance across the Park. Christophersen (2012) used a Park‐wide grid of points with 1‐km spacing to randomly select origin points for sites >1,220 m and within land cover types (Almack et al., 1993) that were dominated by meadows or herbaceous and shrub vegetation. At each site, surveyors chose 2–9 point‐count stations at ~400‐m intervals that optimized visibility of surroundings and coverage of meadows around origin points. The number of stations and size of sites depended on the amount of area suitable for marmots and were limited by the time needed to complete all surveys within 1 morning. Most sites (*n* = 15) had 3–5 stations, suggesting that similar amounts of habitat occurred across sites. Christophersen (2012) surveyed 15 of the 19 sites used in our analysis in 2007 and surveyed the remainder in 2008. In 2016 and 2017, we surveyed all stations within these 19 sites each year, following the survey protocols from Christophersen (2012). We limited our surveys to sites that had marmots in 2007–2008 because many sites were far (~10–25 km) from roads and we could not re‐survey all 31 sites within a summer. All surveys occurred between 12 July and 13 September, except one survey on 26 June 2007. Low correlation of survey date with elevation (*r* = −0.23) indicated that there was no systematic pattern of the order in which sites of various elevations were sampled.

**FIGURE 2:**
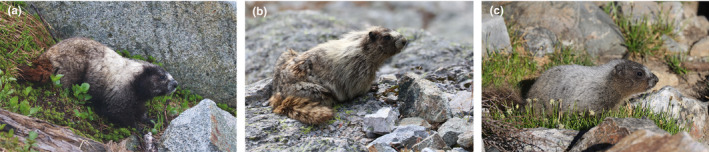
Adult (a), subadult (b), and juvenile (c) hoary marmots (*Marmota caligata*) in North Cascades National Park, Washington, USA

At each station, one surveyor used binoculars to search meadows and boulder fields for 30 min between 07:00 and 12:00 and recorded all marmots seen or heard. When marmots were detected, surveyors used a laser rangefinder to measure the distance to each marmot and classified individuals as adults, juveniles, or subadults (1–2 years old) based on size, color, and behavior, following Braun et al. (2011) (Figure 2). For each survey, we recorded the date, time, cloud cover, temperature, wind, precipitation, and ambient noise (e.g., streams, waterfalls). We described cloud cover to the nearest 10%; wind as low, medium (10–16 km/hr) or high; precipitation as none or light (we did not survey during heavy rain); and noise as low, medium (i.e., limits faint audio detections), or high (i.e., limits audio detections). We surveyed each site once per year, except that Christophersen (2012) surveyed seven of the 19 sites twice in 2008 to assess marmot detectability. Of these, four sites were also surveyed once in 2007. Similarly, we surveyed three sites twice in 2016 because we did not detect marmots on the initial survey; we subsequently detected two marmots on the 2nd survey at one of the three sites. In our analysis, we used only the data from the first survey of any site surveyed >1 time/year to maintain equal survey effort across sites, and we excluded the 2008 data for the four sites also surveyed in 2007.

### 2.3 Weather and snowpack data

As in Johnston et al. (2019), we used metrics of temperature, VPD, precipitation, and snow water equivalent (SWE) from gridded climate surfaces to characterize weather and snowpack dynamics at each site for 2003–2017 (Table 1). Most predictors were based on daily temperature extremes, VPD extremes, and precipitation from PRISM (Parameter‐elevation Relationships on Independent Slopes Model) at 30‐arcseconds (~800‐m) spatial resolution (Daly et al., 2008, 2015). Vapor pressure deficit describes the absolute difference in water vapor from saturation (Johnston et al., 2019) and is newly available in gridded climate data (Daly et al., 2015). Unlike relative humidity, VPD represents the drying effect of air on biota independent of temperature and is ecologically meaningful in its correlation with evapotranspiration (Seager et al., 2015). To characterize snowpack, we used Daymet (Thornton et al., 2017) for daily measures of SWE at 1‐km resolution. These data sources had the finest spatial resolution available for weather in this area.

**TABLE 1:** Weather and snowpack predictors based on PRISM (daily temperature, precipitation and vapor pressure deficit, VPD, at ~800‐m resolution, Daly et al., 2008, 2015) and Daymet (daily SWE at 1‐km resolution, Thornton et al., 2017) in the a priori set of mixed models used to evaluate change in estimated abundances of hoary marmots at North Cascades National Park, Washington, USA, 2007–2008, 2016, and 2017

Predictor	Unit	Definition
Chronic heat	°C	Mean daily maximum temperatures for July–August
Acute heat	days	Number of days with temperature > 25°C for September–August^a^
Growing‐season precipitation	mm	Sum of daily precipitation for July–September
Chronic dryness	kPa	Mean daily minimum VPD for June–September, or November–February
Acute dryness	kPa	Mean daily maximum VPD for June–September, or November–February
Chronic cold	°C	Mean daily minimum temperature for November–February
Acute cold	days	Number of days with temperature < −10°C for September–August
Chronic cold without snowpack	days^b^	Number of days with temperature < 0°C and SWE < 30 mm for September–August
Acute cold without snowpack	°C	Minimum temperature while SWE < 30 mm for September–August
Snowpack duration	days	Number days with SWE > 30 mm for September–August

^a^ Acute heat and cold represented temperature extremes occurring over the entire year because extreme temperatures can occur outside the months used to measure chronic heat, chronic cold, or seasonal VPD.

^b^ Units for chronic cold without snowpack differed from chronic cold because cold exposure without snowpack could occur in fall or spring, when mean daily minimum temperature without snowpack is not sufficiently specific to represent cold exposure. Units for acute cold without snowpack differed from acute cold to represent the most extreme exposure to cold without snowpack.

To index mechanisms of potential stress related to summer weather, we included predictors of growing‐season precipitation, VPD, and exposure to chronic (i.e., mean daily maximum temperatures for July‐August) or acute (i.e., number of days > 25°C) heat. Because the capacity of air to hold vapor increases exponentially with temperature, humid sites (i.e., low minimum VPD) that experience large, within‐day fluctuations in temperature can exhibit high VPD during the warmest time of day, as the temperature warms but the amount of water vapor remains the same. Therefore, we considered the mean of the daily minimum values of VPD for June‐September an index of chronic dryness in summer, whereas the mean of the maximum values represented severity of acute dryness. Minimum and maximum VPD in summer were not completely correlated (*r* = 0.64), indicating that these metrics represented some distinct aspects of moisture. Johnston et al. (2019) included minimum and maximum VPD in summer as separate predictors in a priori models because of their low correlation (*r* = 0.03) at sites of pika surveys, some of which overlapped our marmot sites.

To index mechanisms of potential stress related to winter weather, we included predictors of VPD, snowpack duration, and exposure to acute (i.e., number of days with temperature < −10°C) or chronic (i.e., mean daily minimum temperature from November to February) cold either throughout these 4 months, or specifically at times without snowpack (acute cold = minimum daily temperature; chronic cold = number of days with temperature < 0°C; Table 1). Because snow depths > 20 cm typically provide insulation for subnivean species (Danby & Hik, 2007), we considered snowpack present when Daymet‐estimated SWE ≥ 30 mm. This threshold provided conservative estimates of snowpack duration and cold exposure without snowpack because it corresponded to snow depth of 10 cm for snowpack of moderate density (300 kg/m^3^) that is typical in the Park (Mizukami & Perica, 2008). Correlations of VPD during winter with VPD in summer were low (*r* = 0.28 for VPD minimum and *r* = 0.35 VPD maximum). Correlation between minimum and maximum VPD during winter was high but not complete (*r* = 0.72). As they did for summer VPD, Johnston et al. (2019) reported lower correlation of winter‐time minimum and maximum VPD (*r* = 0.36) at sites of pika surveys, so we treated minimum and maximum separately to create an identical set of a priori models for comparison to the analysis of pika abundances.

### 2.4 Estimating marmot abundance

We used marmot counts and detection distances to estimate detection functions and relative abundances of marmots in Distance 7.3 (Thomas et al., 2010). Although hoary marmots are highly surface‐active during morning in summer (Taulman, 1990), our estimated abundances may not reflect true abundances due to the presence of inactive marmots within burrows during surveys (Corlatti et al., 2017). First, we used conventional distance sampling with the data stratified by site and year to identify an appropriate truncation distance, cut‐point interval, and key function. We truncated marmot detections at 200 m and set the cut‐point interval at 35 m because inclusion of greater distances resulted in detection probabilities < 0.2 and because these values retained 80% of our detections. We selected the hazard‐rate key function with cosine series expansion because it fit the data (*χ* ^2^ = 0.85, *p* = 0.84) and had a lower Akaike information criterion score (AIC, Burnham & Anderson, 2002) than alternative combinations of half‐normal, uniform, and hazard‐rate key functions with series expansions of cosine, simple polynomial, and hermite polynomial.

Next, we used multiple‐covariate distance sampling without adjustments to test the influence of surveyor experience, weather, survey timing, and noise on detection functions. Marmot activity decreases over summer and has a bimodal distribution during the day (Taulman, 1990). Marmots may increase activity during windy or cloudy conditions that reduce heat exposure during summer (Melcher et al., 1990). Detection probabilities for an experienced surveyor who collected data during all years of this study (RGC) did not differ from those of all other surveyors; therefore, we did not include surveyor experience in subsequent analyses. We evaluated an a priori set of models that included univariate models for the effects of Julian date, time of day (minutes after sunrise), cloud cover (sunny or overcast [>80% cover]), temperature, wind (low or high), and noise (low or high) on detection probability. We also tested five models that each combined two of these predictors to represent conditions that could affect detection probabilities (date + time, date + temperature, cloud + wind, noise + wind, and noise + cloud). We combined medium and high categories for wind and noise, because high levels were rare. We did not test precipitation because it only occurred during three of 234 surveys across 78 stations. For subsequent analyses of change in abundance over time and effects of weather, we summed the point estimates of abundance across stations for each site in each year with each model having ∆AIC < 2. Then, we model‐averaged these site‐level abundances by summing the products of each abundance estimate and the Akaike weight of its model (Burnham & Anderson, 2002). Finally, we rounded each model‐averaged estimate of abundance to the nearest whole number.

### 2.5 Analyses of change in marmot abundances

For our 1st objective, we tested whether marmot abundances changed across years and whether changes differed across age classes. To compare marmot abundances at the 19 sites across years, we used zero‐inflated negative binomial models with variance = ɸµ (Hardin & Hilbe, 2018), equal probability of structural zeros for all observations, study site as a random effect to account for the repeated measures of marmot abundance across years, and a fixed effect for year with levels of 2007–2008, 2016, and 2017 in R package *glmmTMB* (Brooks et al., 2017; R Core Team, 2017). We used Type II Wald chi‐square tests in the R package *car* (Fox & Weisberg, 2011) to test significance of factors and Wald *z* tests with Bonferroni corrections to compare factor levels in these mixed‐effects models. Models for continuous data fit abundance and density poorly because estimated abundances were derived from count data. Based on preliminary assessment of a subset of models and AIC scores, zero‐inflated negative binomial models fit the data (KS test *p* = 0.80) better than Poisson, zero‐inflated Poisson, and negative binomial models because of overdispersion and high numbers of zeros (15/57 observations) that were evident in plots of scaled residuals and diagnostic tests for overdispersion, zero inflation, outliers, and goodness of fit in R package *DHARMa* (Hartig, 2020). In a separate analysis, we used a chi‐square test to evaluate differences in the ratio of juvenile, subadult, and adult marmots detected each year.

For our 2nd objective, we evaluated effects of weather and snowpack on marmot abundances with a priori regression models that represented specific hypotheses about mechanistic relationships between life‐history attributes and weather dynamics (Table 2). Models represented our hypotheses that stress from extreme heat or cold reduced marmot abundances and whether VPD, snowpack, or growing‐season precipitation mediated these effects. As we did for our 1st objective, we used zero‐inflated negative binomial models with variance = ɸµ and study site as a random effect to account for the repeated measures of marmot abundance across years in R package *glmmTMB* (Brooks et al., 2017; R Core Team, 2017) to relate marmot abundances at the 19 sites from each year of the study to the summer conditions during the survey or to the winter conditions just prior to each survey. Support for these models would suggest immediate, direct effects of weather on marmot abundances through stress from extreme temperatures. We also tested 1‐year‐lag effects of weather and snowpack by evaluating the same models with predictors based on weather and snowpack conditions from the previous year. Support for lag effects would suggest that exposure to extreme temperatures affected fecundity. We standardized all predictors by subtracting the mean from each observation and dividing by the standard deviation (Norman & Streiner, 2008).

**TABLE 2:** A priori set of mixed models grouped by weather‐based mechanisms used to evaluate change in abundances of hoary marmots in North Cascades National Park, Washington, USA, 2007–2008, 2016, and 2017

Winter models^a^	Summer models
*Cold stress*	*Heat stress*
AC	AH
CC	CH
ACNS	*Summer dryness*
CCNS	AD
AC + ACNS	CD
AC + CCNS	*Summer growing conditions*
CC + ACNS	GSP
CC + CCNS	*Heat stress mediated by dryness*
*Winter dryness*	AH × CD
AD	CH × CD
CD	*Heat stress mediated by growing conditions*
*Cold stress mediated by dryness*	AH × GSP
AC × AD	CH × GSP
AC × CD	*Summer dryness and growing conditions*
CC × AD	AD + GSP
CC × CD	*Heat stress, dryness, and growing conditions*
CCNS × CD	AH + CD + GSP
ACNS × AD	CH + CD + GSP
CCNS × AD	
ACNS × CD	
*Snowpack duration*	
SD	
SD + SD^2^	
*Snowpack duration mediated by dryness*	
SD × AD	
SD × CD	

^a^ Predictors (AH = acute heat, CH = chronic heat, GSP = growing‐season precipitation, CD = chronic dryness, AD = acute dryness, AC = acute cold, CC = chronic cold, ACNS = acute cold without snowpack, CCNS = chronic cold without snowpack, SD = snowpack duration) represented values from the year of each survey. All models included the site as a random factor. Models with interactions (×) included main effects. Additive versions of each model with interactions were included in the a priori set. Additive models for heat stress with dryness included GSP. In addition, we tested this model set with a 1‐year time lag such that predictors that represented conditions from the previous year. Together, there were 93 candidate models for the analyses of marmots including the null model.

Models in the a priori sets included either exclusively summer or exclusively winter factors and had one to three predictors per model (Table 2). Highly correlated predictors (*r* ≥ 0.75) did not appear within the same model. Besides cold exposure and cold exposure without snowpack, we did not include more than one predictor of heat or cold exposure in any model because of high correlations among temperature‐based predictors. We included interactions in several models to determine whether VPD mediated exposure to temperature extremes or effects of snowpack duration. We also tested whether heat stress was mediated by growing‐season precipitation. We evaluated support for hypotheses based on the Akaike information criterion for small sample sizes (AIC*_c_*) and Akaike weights (Burnham & Anderson, 2002). We considered models within two AIC*_c_* units of the top model similarly plausible.

## 3 RESULTS

### 3.1 Estimation of marmot abundances

We counted 377 marmots, of which 300 were ≤200 m from point‐count stations and used to estimate abundance (Table 3). The top‐ranked model for estimating marmot abundance indicated that detection probability was higher during high (*P_a_* = 0.52, SE = 0.19) versus low (*P_a_* = 0.28, SE = 0.14) wind. Three other models had ∆AIC < 2 and indicated that detection probabilities (a) decreased over summer (∆AIC = 0.05); (b) increased with cloud cover and wind (∆AIC = 0.69); or (c) decreased with noise but increased with cloud cover (∆AIC = 1.28).

**TABLE 3:** Total counts, estimated abundance (*N*, 95% CI), counts of juveniles:subadults:adults (J:S:A), and the number of sites and stations with detections (all detections: detections ≤ 200 m) of hoary marmots at 78 point‐count stations across 19 sites in North Cascades National Park, Washington, USA, 2007–2008, 2016, and 2017

Year	Total count	Count ≤ 200 m	Abundance ≤ 200 m^a^	J:S:A^b^	Sites	Stations
2007–2008	228	174	543, 396–744	24:33:141	19:18	55:46
2016	60	46	142, 86–233	13:02:34	16:12	29:22
2017	91	80	293, 195–441	16:10:59	14:12	34:32

^a^ Abundances are model‐averaged estimates from models with data stratified only by year and ΔAIC < 2 based on distance sampling and detection functions that accounted for survey conditions.

^b^ Ratios represent all marmots detected. Ratios for marmots detected ≤ 200 m are reported in the text.

### 3.2 Change in marmot abundances across years

**FIGURE 3:**
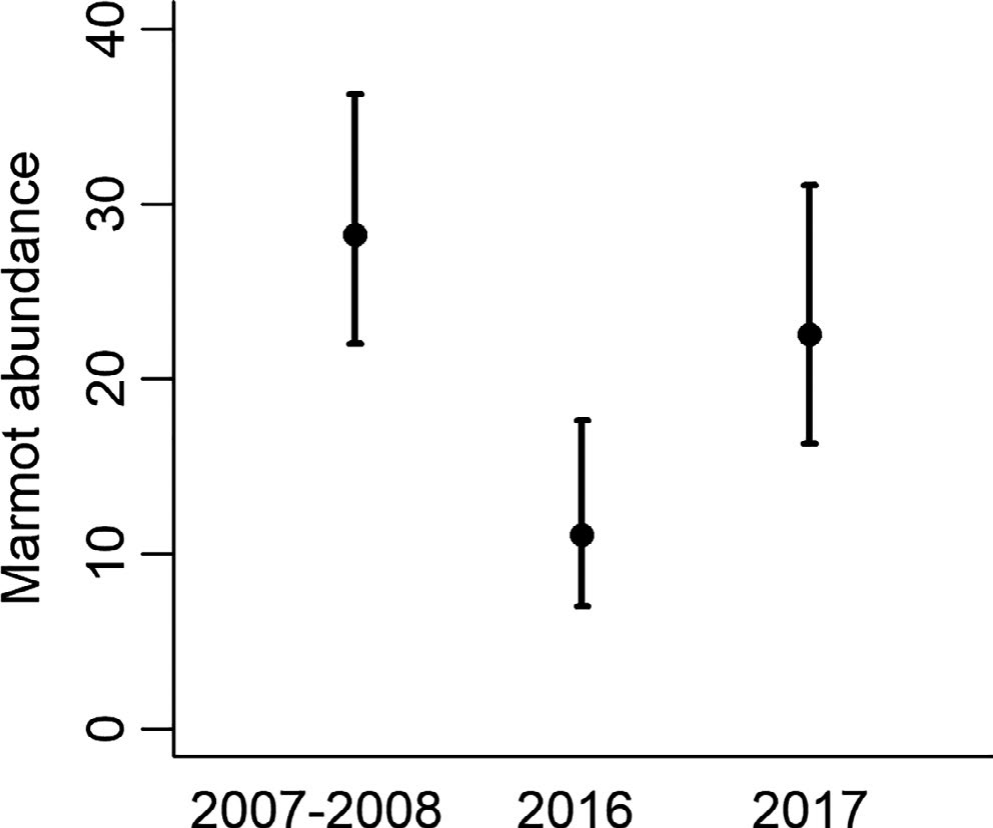
Mean abundance and 95% CIs of hoary marmots at 19 sites in North Cascades National Park, Washington, USA, 2007–2008, 2016, and 2017. Marmot abundance at 78 stations across 19 sites was estimated with distance sampling and detection functions. Mean abundance at sites for each year of survey was estimated subsequently with a negative binomial model with mixed effects and that accounted for zero inflation

Marmot abundances differed across years (*χ* ^2^_2_ = 16.3, *p* < 0.001) and were higher in 2007–2008 than in 2016 (*z* = 4.0, Bonferroni *p* < 0.001) but not in 2017 (*z* = 1.3, Bonferroni *p* = 0.58, Table 3, Figure 3). Abundances were lower in 2016 than in 2017 (*z* = 2.9, Bonferroni *p* = 0.011). The ratio of juvenile, subadult, and adult marmots detected ≤ 200 m from observers differed across years (*χ* ^2^_4_ = 10.9, *p* = 0.028, 2007–2008 = 20:33:101, 2016 = 11:2:24, 2017 = 16:10:51) mostly because we detected more juveniles but fewer subadults than expected in 2016. Results of age‐class comparisons were similar with marmot detections > 200 m included (Table 3). From 2007–2008 to 2016, estimated marmot abundance decreased at 17 of 19 sites, and marmot detections, including those > 200 m, decreased at all sites.

### 3.3 Effects of weather and snowpack on marmot abundance

**FIGURE 4:**
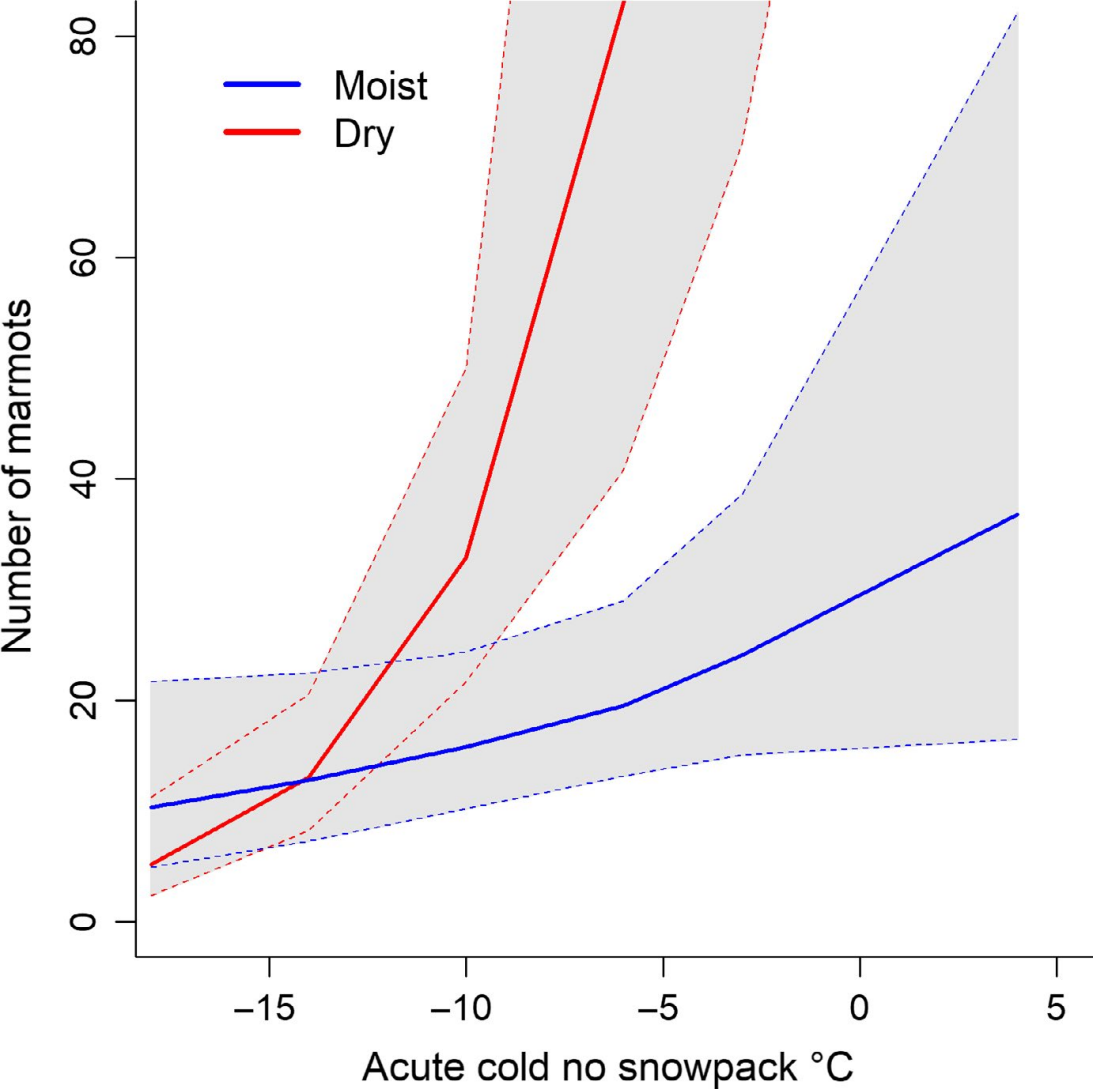
Interactive effects of chronic dryness and acute cold exposure without snowpack on marmot abundance based on predicted values (solid lines) and 95% CIs (dashed lines) from the top‐ranked model for abundance of hoary marmots at North Cascades National Park, Washington, USA, 2007–2008, 2016, and 2017. Mean intercepts for the random effects of sites were used for each prediction of marmot abundance. Low (i.e., moist; blue lines) and high (i.e., dry; red lines) vapor pressure deficit (VPD) prediction lines are based on the 10th‐ and 90th‐percentile values of mean daily minimum VPD observed during winter. Predictions cover the approximate range of values for the combination of chronic dryness and acute cold without snowpack observed during the study

The top‐ranked model that related marmot abundance to weather and snowpack included the interaction of chronic dryness in winter (i.e., mean daily minimum VPD) and acute cold exposure without snowpack from the year prior to survey (i.e., 1‐year lag effect; Table 4). The interaction indicated that decreases in marmot abundance following exposure to extreme cold without snowpack were especially severe under chronically dry conditions (Figure 4). Under moist conditions, cold exposure led to little change in marmot abundances, which were typically low. The second‐ranked model (ΔAIC*_c_* = 0.6) also had predictors that represented 1‐year‐lag effects and indicated that marmot abundances increased following cold winter conditions but decreased after exposure to extreme cold without snowpack. All other models had ΔAIC*_c_* > 2. The sum of Akaike weights for models that represented winter weather and snowpack dynamics was 0.90. Models that included predictors based on winter temperature had a cumulative weight = 0.81; those with VPD in winter had a sum = 0.47; and those with predictors representing 1‐year‐lag effects of winter weather had a sum = 0.82. Models indexing summer weather ranked far below those with winter‐weather and snowpack predictors; specifically, the highest‐ranked model based on summer weather had ΔAIC*_c_* = 4.6 and Akaike weight = 0.02.

**TABLE 4:** Differences in Akaike's information criterion for small sample sizes (ΔAIC*_c_*), model weights (*w*_i_), and model coefficients (SE) for models with ΔAIC*_c_* < 4 plus the null model in the a priori set of mixed models used to evaluate change in estimated abundances of hoary marmots based on weather and snowpack dynamics at North Cascades National Park, Washington, USA, 2007–2008, 2016, and 2017

Model^a^	*β*_1_	*β*_2_	*β*_3_	ΔAIC*_c_*	*w*_i_
CD × ACNS	0.19(0.10)	0.59(0.11)	0.18(0.07)	0	0.25
CC + ACNS	−0.23(0.10)	0.22(0.08)		0.60	0.18
CC + CCNS	−0.36(0.09)	−0.36(0.19)		2.23	0.08
ACNS	0.31(0.07)			3.08	0.05
AC + ACNS	0.11(0.08)	0.25(0.08)		3.71	0.04
CD + ACNS	0.13(0.10)	0.38(0.09)		3.99	0.03
CC	−0.38(0.10)			3.99	0.03
Null				14.31	<0.001

^a^ CD = chronic dryness in winter, ACNS = acute cold exposure without snowpack, CC = chronic cold exposure, CCNS = chronic cold exposure without snowpack, AC = acute cold exposure. All models in this table had site as a random factor and predictors that represented winter weather 1 year prior to the marmot survey (i.e., 1‐year‐lag effects). Predictors were standardized to a normal distribution with a mean of 0 and standard deviation of 1. The top‐ranked model included main effects of each predictor with the interaction (*β*_3_).

## 4 DISCUSSION

Results of this study supported our hypotheses that marmot abundances (a) decreased from baseline levels following the 2015 snow drought, (b) decreased with increasing cold exposure without snowpack, and (c) were influenced more by weather in winter than summer. We also found that the effects of cold exposure were mediated by vapor pressure deficit (VPD) and that changes in abundance varied across age classes. This study adds to growing evidence that VPD, an index of atmospheric moisture and the drying effect of air, is an important aspect of climate for biota that can improve understanding of ecosystem dynamics. As with pikas in North Cascades National Park (Johnston et al., 2019), the importance of winter weather to marmots contrasts with more‐common hypotheses that cold‐adapted species are most affected by heat exposure or changes in growing‐season conditions under increasing temperatures (Rézouki et al., 2016). Our results could have important implications for the ecology and conservation of montane wildlife because snow‐drought frequency (Marlier et al., 2017) and VPD (Ficklin & Novick, 2017) are predicted to increase with global mean temperature.

### 4.1 Evaluating alternative hypothesized mechanisms

The widespread decrease in marmot abundances that occurred between 2007 and 2016 was suggestive of severe, wide‐reaching effects from the 2015 snow drought. Although we lacked estimates of marmot abundances in 2014 and 2015 to more directly link the 2015 snow drought to this change in marmot abundances, our top model that explained changes in marmot abundances over time was composed of predictors for winter weather that were related to snow drought and was consistent with lagged effects of winter stress on marmot abundances reported previously (Patil et al., 2013; Rézouki et al., 2016; Tafani et al., 2013). Chronic dryness peaked during the snow drought, and exposure to acute cold without snowpack can be severe during snow drought (Appendix S1, Table S3, Figure S1). The 74% decrease in marmot abundance from 2007 to 2016 is much greater than the largest fluctuations observed in other multi‐year studies on hoary marmots in Yukon Territory, Canada (6 years; 55% change; Patil, 2010), and alpine marmots (15 years; 33% change; Stephens et al., 2002). However, a 75% change in abundance of yellow‐bellied marmots (*M. flaviventer*) occurred within a 40‐year study (Oli & Armitage, 2004), which showed that at least some marmot species can experience and withstand large fluctuations over time. Hoary marmots are not prone to irruptive population dynamics because they live up to 12 years and produce ≤ 4 young per litter, biennially (Barash, 1989). Metapopulation dynamics might have contributed to decreases in abundance during our study (Griffin et al., 2008), but we did not observe concomitant increases in abundance or detections of marmots at stations with few or no previous detections in 2007–2008. Although little is known about movement and dispersal of hoary marmots (Kyle et al., 2007; Patil, 2010), other marmot species exhibit limited movement (Griffin et al., 2009) and continuous, long‐term occupancy of high‐quality habitats (Blumstein et al., 2006; Ozgul et al., 2006). We could not evaluate other factors like predation (Bryant & Page, 2005; Witczuk et al., 2013) and disease (Vander Haegen et al., 2018) that could have contributed to the decrease in marmot abundance between 2007 and 2016.

Our results provide evidence that chronically dry conditions in winter exacerbate physiological stress on hibernating marmots from exposure to extreme cold without insulating snowpack (Barash, 1973; Patil et al., 2013; Rézouki et al., 2016; Tafani et al., 2013). Slight increases in cold exposure can reduce marmot fitness because oxygen consumption (i.e., energy expenditure) of hibernating marmots at ambient temperatures of 0°C is four times higher than at 5°C (Arnold et al., 1991). Although severe cold exposure without snowpack had occurred in recent years, the combination of cold temperatures with extraordinarily dry air in 2015 was unusual and may have compounded stress (Appendix S1, Table S3, Figure S1). Counter to our prediction, cold stress was not exacerbated by moisture (i.e., low VPD); instead, negative effects of chronic dryness were suggestive of dehydration during hibernation. Marmots can be sensitive to moisture, as evidenced by reduced metabolic rates of marmots under water restriction (Armitage et al., 1990). Moisture of subnivean air is typically high and decoupled from surface conditions because of snow cover (Aitchison, 2001); however, Armitage et al. (1990) characterized marmot hibernacula as xeric environments. The short duration of snowpack in 2015 may have increased exposure to cold, dry conditions for marmots while they were still hibernating and inflicted physiological stress through increased arousals from dehydration (Ben‐Hamo et al., 2013) and cold exposure. This stress may have led to reproductive failure, which might explain our observations of fewer subadults one year after the snow drought (Armitage & Blumstein, 2002; Patil et al., 2013; Rézouki et al., 2016). In addition, high mortality of juvenile marmots during low‐snow years has been attributed to cold stress (Barash, 1973; Cordes et al., 2020; Patil et al., 2013), which might further explain low numbers of subadults that were 2 years old in summer 2016. Juvenile marmots may be especially vulnerable to cold stress during hibernation because of their small size, and poor juvenile survival could lead to low survival in subsequent years for young marmots that depend on social hibernating for thermal regulation (Rézouki et al., 2016).

Alternatively, warm weather and snow‐free conditions, such as those during the 2015 snow drought (Marlier et al., 2017, Appendix S1, Table S3, Figure S1), can destabilize temperatures in burrows and lead to early emergence. Arousals of hoary marmots from hibernation has been correlated with snow depth (Patil et al., 2013), and increased frequency of arousals may indicate stressful burrow conditions. Energy expenditure of active marmots is significantly higher than hibernating marmots (Armitage et al., 1990, 2003), and temperature influences the specific timing of emergence. For example, emergence of yellow‐bellied marmots in Colorado is correlated with minimum temperatures in April and occurred 23 days earlier in the 1990's relative to the 1970's (Inouye et al., 2000). However, early emergence of some populations of yellow‐bellied marmots has coincided with increases in fitness that were attributed to longer growing seasons (Ozgul et al., 2010). The consequences of early emergence for hoary marmots probably depend on availability and nutritive value of forage at the time of emergence. The greater support for acute cold stress without insulating snowpack compared to snowpack duration suggests that early emergence was less likely a driving mechanism of marmot abundance in the Park.

Another alternative mechanism related to snow drought might involve reduced quality and quantity of forage for marmots from early snowmelt and dry air, leading to lower availability of nutritious forage in late summer. Meeting fat requirements for hibernation is paramount to marmot survival (Karels & Hik, 2003; Ozgul et al., 2010; Schwartz & Armitage, 2005; Van Vuren & Armitage, 1991). Alpine plant productivity depends on timing of snowmelt and soil moisture (Billings & Bliss, 1959; Körner, 2003). Dry air (i.e., high VPD) reduces soil moisture and causes plants to minimize water loss by closing stomata, which inhibits plant productivity (Ficklin & Novick, 2017; Wilson & Guitierrez, 2012) and likely affects forage quantity and quality for marmots. Furthermore, Frase and Armitage (1989) observed marmots moving from dry to moist areas during times of water stress, which suggests that marmots are sensitive to micro‐site variation in moisture and plant quality.

To assess the importance of forage more directly, we performed a post hoc assessment of models that represented phenology and productivity of vegetation (Appendix S2). Results indicated that models with predictors from time series of the normalized difference vegetation index (NDVI) from the Moderate‐Resolution Imaging Spectroradiometer (MODIS, Johnston et al., 2018) to represent hypotheses about effects of vegetation dynamics on marmots were not competitive with our weather‐based models. Therefore, we suggest that physiological stress from cold exposure and dry air likely affected marmots more strongly than altered forage conditions did. Intensive tracking studies of marmots with in situ, fine‐scale measurements of vegetation and environmental conditions at marmot‐relevant scales are needed to further discriminate mechanistic relationships, though such methods can be time‐ and equipment‐intensive for remote, wilderness contexts such as ours (Johnson et al., 2008).

### 4.2 Inter‐specific differences in responses to snow drought

Winter weather and snowpack dynamics explained far more variation in pika (Johnston et al., 2019) and marmot abundances than did summer weather in the Park. Whereas most studies have implicated heat exposure as the mechanism of range retraction for *O. princeps* in the southern portion of its geographic range (Beever et al., 2003, Wilkening et al., 2011, Stewart et al., 2015, Beever et al., 2016, but see Beever et al., 2011), winter weather dynamics were resoundingly more important in North Cascades National Park. Like pikas, marmots are poorly adapted to heat exposure (Armitage, 2013); nonetheless, our models involving heat stress explained relatively little variation in marmot abundances. The strong effect of winter weather on hoary marmots in the Park is consistent with the seasonal drivers of abundance at the northern extent of their range in Canada (Patil et al., 2013). The importance of winter dynamics may reflect the region's maritime influence and comparatively milder climate characterized by fewer hot and cold extremes (Varner & Dearing, 2014). At the species’ northern‐range extent, early snowmelt reduces survival of hoary marmots, particularly among young marmots in hibernation, most likely via lack of insulation from cold temperatures (Barash, 1989; Patil et al., 2013). Both species have shown 1‐year lags in their responses to winter weather, indicative of effects on survival or recruitment following stressful, but sub‐lethal conditions (Johnston et al., 2019; Patil et al., 2013). Like pikas, marmots resorb embryos when spring conditions are stressful (Armitage & Blumstein, 2002). Winter weather has induced similar time lags in population dynamics due to strong effects on survival or fecundity for other mammalian herbivores, including Soay sheep (*Ovis aries*, Coulson et al., 2000), red deer (*Cervus elaphus*, Forchhammer et al., 1998), and reindeer (*Rangifer tarandus*, Bårdsen et al., 2010).

Our top models for marmots were remarkably similar to those of pikas (Johnston et al., 2019) in indicating the strong influence of winter weather on populations and the weather dynamic of importance: the interaction of chronic dryness and snowpack duration (pikas) or acute cold without snowpack (marmots), each with 1‐year‐lag effects. Like pikas, marmot abundances were lower following warm, dry conditions during winter. However, the near‐universal increase in relative abundance of pikas at elevations above 1,400 m after winter 2015 where marmot abundances were low is a striking contrast that suggests different mechanisms from the same weather factors acting on these species. Whereas our study area is near the southern geographic limit for marmots, it is near the northern limit for pikas. This region receives more snow‐fall than any other in the range of *O. princeps*, and the snow drought appeared to increase pika abundances at elevations > 1,400 m due to increased foraging opportunities associated with early snow melt, but marmots did not show such benefits from increased forage. Evidence for negative effects of cold exposure without snowpack was much stronger for marmots than pikas. Although marmots conserve warmth by hibernating socially, these benefits can be overwhelmed by extreme cold (Arnold, 1988; Rézouki et al., 2016). Pikas are active throughout winter and may mitigate weather‐related stress by (a) moving into microrefugia (e.g., beneath snow drifts, deep talus, large rocks with more‐stable conditions due to their greater thermal inertia) to avoid cold exposure (Beers, 2016), or (b) drinking water or eating snow as needed, whereas marmots have less behavioral plasticity during hibernation. Talus matrices used by pikas have thermal dynamics (Millar et al., 2014) that may provide better insulation from cold than marmot burrows without snow cover. Furthermore, pikas may be better suited to meet changing energy requirements during winter because of their reliance on haypiles rather than fat for winter survival (Wunder, 1992). Regardless of mechanism, the apparent increase in pikas simultaneous to decreases in marmots at high elevations suggests nuanced differences in responses to weather and climate in these sympatric species. These species might share sensitivity to extreme cold and dry air in the absence of snowpack, but the consequences of these factors may differ between species within areas of sympatry.

Warm winters that lead to snow droughts are expected to occur more frequently over the next century and may lead to further declines in marmot abundance. Long‐term demographic studies on hoary marmots are needed to further discriminate mechanisms related to weather, snowpack, forage, predators, and disease. Vapor pressure deficit interacted with cold exposure to influence on marmots and shows promise as a new predictor for animal abundance. Despite similar predictors of abundance with pikas, marmots appeared to respond differently to winter weather in areas of sympatry, which could have significant implications for conservation strategies that use one of these species as an indicator for the other. Differential responses to weather between pikas and marmots could lead to their geographic divergence as global change continues.

## CONFLICT OF INTEREST

None declared.

## AUTHOR CONTRIBUTIONS

**Aaron N. Johnston:** Conceptualization (lead); data curation (supporting); formal analysis (lead); funding acquisition (lead); investigation (lead); methodology (lead); project administration (lead); writing‐original draft (lead); writing‐review & editing (lead). **Roger G. Christophersen:** Conceptualization (supporting); data curation (lead); investigation (supporting); methodology (supporting); writing‐review & editing (supporting). **Erik A. Beever:** Conceptualization (supporting); formal analysis (supporting); funding acquisition (supporting); investigation (supporting); methodology (supporting); writing‐original draft (supporting); writing‐review & editing (supporting). **Jason I. Ransom:** Conceptualization (supporting); data curation (supporting); investigation (supporting); methodology (supporting); writing‐review & editing (supporting).

## Supporting information

Appendix S1‐S2

## Data Availability

Data are available in the ScienceBase Catalog at https://doi.org/10.5066/P9K0QEDT.
