# Supplementary material for: Freezing in a warming climate: Marked declines of a subnivean hibernator after a snow drought
Source: Ecol Evol. 2020 Dec 29;11(3):1264–79. doi: 10.1002/ece3.7126 (PMC7863385; doi:10.1002/ece3.7126)
Supplement: Supplementary file 1 — Appendix S1‐S2 [file ECE3-11-1264-s001.docx]

Appendix S1. Supplemental tables and figures for the analysis of hoary marmot responses to weather and snowpack dynamics in North Cascades National Park, Washington, USA, 2007-2008, 2016, and 2017.

Table S1. Effect estimates from univariate models for predictors included in the *a priori* set of mixed models used to evaluate change in estimated abundances of hoary marmots based on weather and snowpack dynamics in North Cascades National Park, Washington, USA, 2007-2008, 2016, and 2017.

| Predictor^a^ | Season | *β* | SE |
| --- | --- | --- | --- |
| Acute heat (days) | Summer | -0.394* | 0.025 |
| Chronic heat (°C) | Summer | 0.114 | 0.112 |
| Growing-season precipitation (mm) | Summer | -0.371* | 0.126 |
| Chronic dryness (kPa) | Summer | -0.098 | 0.088 |
| Acute dryness (kPa) | Summer | 0.042 | 0.115 |
| Acute cold (days) | Winter | 0.244* | 0.080 |
| Chronic cold (°C) | Winter | -0.381* | 0.095 |
| Acute cold without snowpack (°C) | Winter | 0.308* | 0.074 |
| Chronic cold without snowpack (days) | Winter | -0.443* | 0.198 |
| Snowpack duration (days) | Winter | 0.366* | 0.108 |
| Chronic dryness (kPa) | Winter | -0.090 | 0.090 |
| Acute dryness (kPa) | Winter | -0.107 | 0.104 |

^a^All predictors were standardized to a normal distribution with a mean of 0 and standard deviation of 1 for the analysis. Estimates are for 1-year lag effects.

^*^*P* < 0.05 that *β* ≠ 0 beta is not equal to zero.

Table S2. Coefficient signs of predictors and sums (*w*+) and means () of Akaike weights of models with each predictor from the *a priori* set of mixed models used to evaluate change in estimated abundances of hoary marmots based on weather and snowpack dynamics in North Cascades National Park, Washington, USA, 2007-2008, 2016, and 2017.

| Predictor | Season | *w*+^a^ |  | Sign^b^ |
| --- | --- | --- | --- | --- |
| Acute heat (days) | summer | 0.045 | 0.009 | - |
| Chronic heat (°C) | summer | 0.049 | 0.010 | - |
| Growing-season precipitation (mm) | summer | 0.099 | 0.012 | - |
| Chronic dryness (kPa) | summer | 0.024 | 0.005 | 2+,1- |
| Acute dryness (kPa) | summer | 0.002 | 0.001 | 1+,1- |
| Acute cold (days) | winter | 0.054 | 0.008 | + |
| Chronic cold (°C) | winter | 0.354 | 0.051 | - |
| Acute cold without snowpack (°C) | winter | 0.586 | 0.084 | + |
| Chronic cold without snowpack (days) | winter | 0.090 | 0.015 | - |
| Snowpack duration (days) | winter | 0.039 | 0.007 | + |
| Chronic dryness (kPa) | winter | 0.323 | 0.029 | 4+,2- |
| Acute dryness (kPa) | winter | 0.070 | 0.006 | 1+,5- |

^a^Signs and Akaike weights are from models with 1-year lags because no model without the time lag had a weight >0.02.

^b^Coefficient signs were consistent across all models, unless the number of models with positive and negative signs is listed (e.g., 2+,1-, which indicated that 2 models had that predictor with a positive coefficient, whereas 1 model had it with a negative coefficient). Signs represent results from models with main effects only.

## Winter weather anomalies

We used a binary variable that identified observations from 2015 in linear mixed-effects models from R package *lme4* (Bates et al. 2015) to compare weather and snowpack conditions during the winter of 2014-2015 to other years in our data set from 2003 through 2017. Our evaluation included weather 5 years prior to the first marmot surveys in 2007 to describe conditions that could have influenced abundances in 2007 and increase the number of years for comparison of weather to 2015. We evaluated differences based on Type II Wald *F* tests with Kenward-Roger estimates for degrees of freedom (Kenward and Roger 1997). Study site was included in all models as a random factor to account for the repeated measures.

As measured by gridded climate values, the winter of 2015 was anomalous to other years during 2003 through 2017 at our sites based on snowpack, mean daily minimum VPD in winter, and chronic cold (Appendix S1: Figure S1; Table S3). Specifically, snowpack duration and air moisture were at their lowest levels in winter 2015, across the 15-year period. Our measures of chronic cold suggest 2015 was the warmest winter during the study period. Despite the warm winter (or perhaps due to it and the consequent lack of an insulating snow ‘blanket'), cold exposure without snowpack in 2015 was ranked 4^th^-most severe of the 15 years in our dataset.

Table S3. Results of mixed models (*β*, SE, *F*) that compared winter weather and snowpack in 2015 with other years from 2003–2017. In addition, the far-right column indicates rank of 2015 versus all 14 other years at survey sites for hoary marmots, in which highest ranking years had the shortest snowpack duration, driest air, and the most severe cold exposure in North Cascades National Park, Washington, USA.

| Response variable | *β* | SE | *F* | Rank |
| --- | --- | --- | --- | --- |
| Snowpack duration (days) | -98.1 | 13.2 | 56 | 1 |
| Chronic dryness (kPa) | 0.299 | 0.031 | 92 | 1 |
| Acute dryness (kPa) | 0.586 | 0.110 | 28 | 2 |
| Chronic cold (°C) | 2.60 | 0.23 | 127 | 15 |
| Acute cold (days) | -13.8 | 2.5 | 30 | 14 |
| Chronic cold without snowpack (days) | 9.60 | 6.23 | 2.4 | 4 |
| Acute cold without snowpack (°C) | -3.73 | 1.56 | 5.7 | 4 |


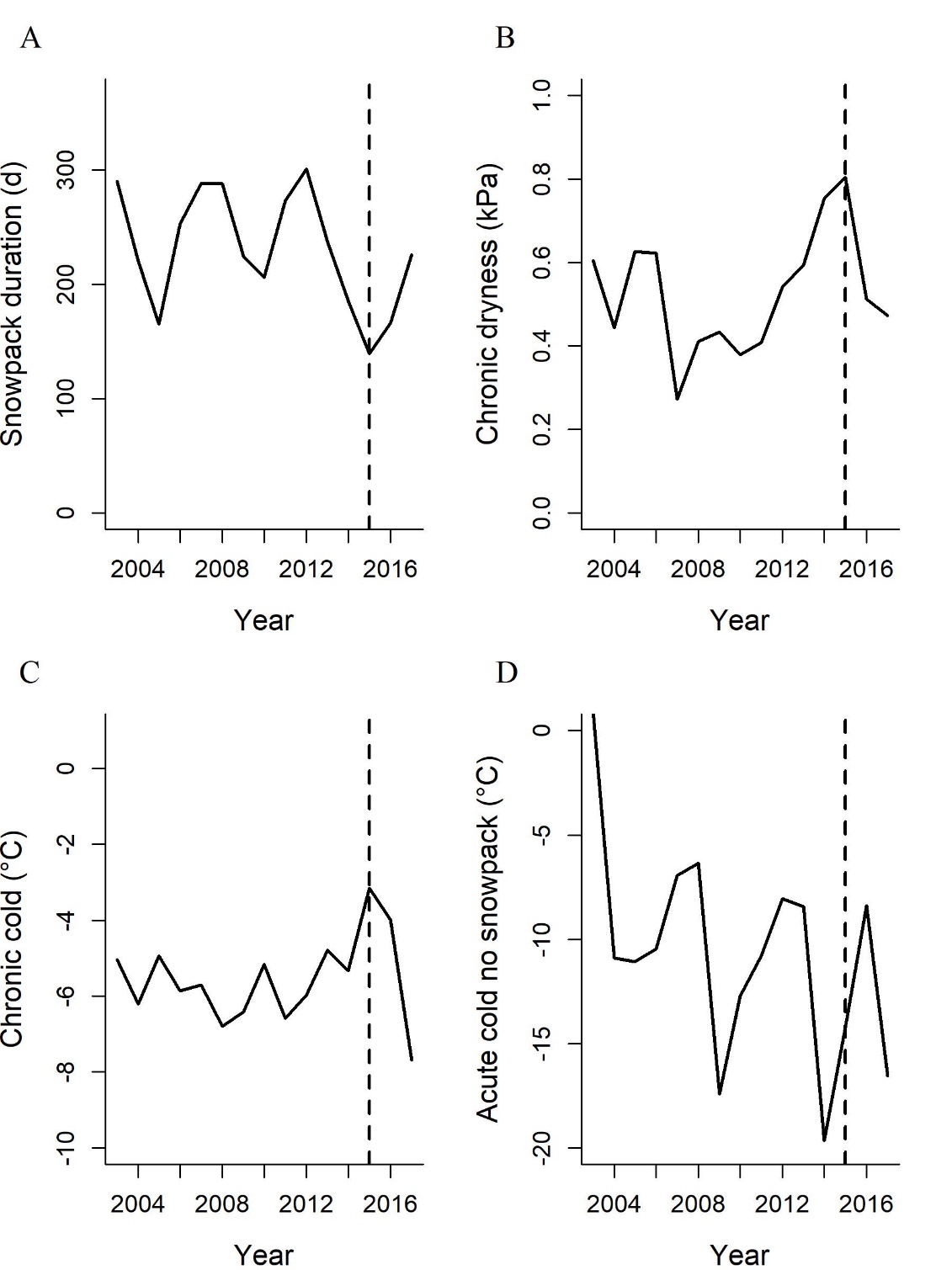


Figure S1. Annual snowpack duration (A), chronic dryness (mean daily minimum vapor pressure deficit) in winter (B), chronic cold (C), and acute cold without snowpack (D) during 2003–2017 averaged over all 78 stations from 19 sites that were used to survey hoary marmots in North Cascades National Park, Washington, USA, 2007-2008, 2016, and 2017. The vertical dashed line marks the record-low snowpack in 2015.

**Appendix S2.** Methods and results of *post hoc* analyses of hoary marmot abundance in North Cascades National Park, Washington, USA, 2007–2017.

## Methods

We conducted *post hoc* analyses to 1) evaluate the influence of forage availability and quality, and 2) compare vegetation conditions in 2015 to other years. For the 1^st^ objective, we tested a suite of *post hoc* models (Appendix 2: Table S1) to discriminate direct (i.e., physiological stress) and indirect (Ockendon et al. 2014) mechanisms (i.e., forage availability) of effects of weather and snowpack that were implied by our top models in our *a priori* analyses (Table 2). Like the *a priori* analyses, we modeled estimated abundances of marmots as a function of vegetation-based predictors, including site as a random factor in zero-inflated negative binomial models. Predictors reflected aspects of the time series of the normalized difference vegetation index (NDVI) in imagery from the Moderate Resolution Imaging Spectroradiometer (MODIS) on the Terra satellite (Jenkerson et al. 2010, Johnston et al. 2018). We used measures of productivity (i.e., cumulative NDVI, maximum NDVI, NDVI amplitude) and phenology (start of season, end of season, season duration) that were calculated from the NDVI curve for each year of the study period. These USGS-calculated metrics were available at https://doi.org//10.5066/F7PC30G1 (accessed 20 April 2019). We hypothesized that marmot abundances would increase with forage availability, which we hypothesized in turn would increase with growing-season productivity and duration. Early senescence in August or September could limit forage availability and prevent marmots from accumulating enough fat for winter survival. Because our *a priori* analysis indicated that dryness (i.e., VPD) mediated other stressors, we included models with the interactions between dryness and metrics of phenology or productivity as a complement to the suite of models without these predictors. Moisture, for example, could mediate the effect of early senescence if moisture increases quantity and quality of forage for marmots. We only evaluated models with predictors that represented conditions from the summer prior to each year’s survey because evidence for 1-year-lag effects of weather on marmots was strong in the *a priori* analysis.

For the 2^nd^ objective, we used linear mixed-effects models from R package *lme4* (Bates et al. 2015) to compare phenology and productivity during 2015 to other years in our data set from 2003 through 2017. These models used the measure of phenology or productivity at each site in each year as the response predicted by a binary variable that identified observations from 2015 and a random factor for site. Our evaluation included phenology and productivity 5 years prior to the first marmot surveys in 2007 to describe conditions that could have influenced abundances in 2007 and increase the number years for comparison of vegetation to 2015. We evaluated differences based on Type II Wald F tests with Kenward-Roger estimates for degrees of freedom (Kenward and Roger 1997). Study site was included in all models as a random factor to account for the repeated measures.

## Results

For the 1^st^ objective, the top-ranked model based on vegetation indicated that marmot abundances decreased the year after high cumulative NDVI (i.e., productivity) and increased with later dates for start of spring. Although this model included the interaction of these predictors, only additive effects were evident (interaction coefficient *z* = 0.83, *P* = 0.40). We did not include the combination of these predictors without interaction in our *a priori* model set. This model was not competitive with the top-ranked model in the *a priori* set with predictors that represented weather and snowpack (ΔAIC*_c_* = 4.15; Table S2). No vegetation model was within 2 AIC*_c_* of the top-ranked model based on vegetation. Results of univariate analyses (Appendix 2: Table S3) did not support our *post hoc* hypotheses about the importance of forage availability and quality to marmots. Marmot abundances decreased with increasing season length and productivity.

For our 2^nd^ objective, vegetative productivity based on cumulative NDVI was highest and start of season was earliest in 2015 compared to other years from 2003 to 2017 (Appendix 2: Table S4, Figure S2). Although 2015 had the earliest start of season, longest season duration, and the highest cumulative NDVI, that year was typical in terms of the end of the season, maximum NDVI, and NDVI amplitude.

Table S1. *Post hoc* set of mixed models used to evaluate change in estimated abundances of hoary marmots based on plant phenology and productivity in North Cascades National Park, Washington, USA, 2007-2008, 2016, and 2017.

| Model^a^ | Model |
| --- | --- |
| 1. Start of Season | 15. Cumulative NDVI x Chronic Dryness |
| 2. Start of Season + Start of Season^2^ | 16. Cumulative NDVI x Acute Dryness |
| 3. End of Season | 17. Maximum NDVI |
| 4. Season Duration | 18. Maximum NDVI x Start of Season |
| 5. Start of Season x Chronic Dryness | 19. Maximum NDVI x Season Duration |
| 6. End of Season x Chronic Dryness | 20. Maximum NDVI x End of Season |
| 7. Season Duration x Chronic Dryness | 21. Maximum NDVI x Chronic Dryness |
| 8. Start of Season x Acute Dryness | 22. Maximum NDVI x Acute Dryness |
| 9. End of Season x Acute Dryness | 23. NDVI Amplitude |
| 10. Season Duration x Acute Dryness | 24. NDVI Amplitude x Start of Season |
| 11. Cumulative NDVI | 25. NDVI Amplitude x Season Duration |
| 12. Cumulative NDVI x Start of Season | 26. NDVI Amplitude x End of Season |
| 13. Cumulative NDVI x Season Duration | 27. NDVI Amplitude x Chronic Dryness |
| 14. Cumulative NDVI x End of Season | 28. NDVI Amplitude x Acute Dryness |

^a^Predictors represented conditions from the year before each mammal survey. All models included a random factor for site. Normalized difference vegetation index = NDVI.

Table S2. Differences in Akaike’s information criterion for small sample sizes (ΔAIC*_c_*), model weights (*w*_i_), and model coefficients (SE) for vegetation-based models with ΔAIC*_c_* < 4 of the top-ranked vegetation model plus the null model from the set of mixed models used to evaluate change in estimated abundances of hoary marmots based on plant phenology and productivity in North Cascades National Park, Washington, USA, 2007-2008, 2016, and 2017. Model weights and ΔAIC*_c_* are based on all models including those of the *a priori* set for weather and snowpack.

| Model^a^ | *β*_1_ | *β*_2_ | *β*_3_ | ΔAIC*_c_* | *w*_i_ |
| --- | --- | --- | --- | --- | --- |
| NDVI_c_ x SOS | -0.39(0.14) | 0.40(0.14) | 0.12(0.15) | 4.15 | 0.029 |
| SOS | 0.37(0.14) |  |  | 7.22 | 0.006 |
| AMP x SOS | -0.29(0.13) | 0.57(0.15) | 0.05(0.14) | 7.28 | 0.006 |
| Null |  |  |  | 14.3 | <0.001 |

^a^NDVI_c_ = cumulative normalized difference vegetation index, SOS = start of season, AMP = NDVI amplitude. All models in this table had site as a random factor and predictors that represented phenology and productivity from the year prior to the marmot survey (i.e., 1-year lag effects). Predictors were standardized to a normal distribution with a mean of 0 and standard deviation of 1. The top-ranked model included main effects of each predictor with the interaction (*β*_3_).

Table S3. Effect estimates from univariate models with standardized predictors of vegetative phenology and productivity from the summer prior to hoary marmot surveys and the mean Akaike weight () for each model containing each of these predictors, based on weights from the combined *a priori* and *post hoc* model sets used to evaluate change in estimated abundances of hoary marmots based on weather and vegetation in North Cascades National Park, Washington, USA, 2007-2008, 2016, and 2017.

| Predictor | *β* | SE |  |
| --- | --- | --- | --- |
| Start of season (Julian day) | 0.373* | 0.137 | 0.007 |
| End of season (Julian day) | 0.021 | 0.113 | 0.001 |
| Season duration (days) | -0.263* | 0.105 | 0.001 |
| Cumulative NDVI^a^ | -0.424* | 0.172 | 0.008 |
| Maximum NDVI | -0.065 | 0.100 | 0.003 |
| NDVI amplitude | 0.011 | 0.101 | 0.001 |

^a^Normalized difference vegetation index = NDVI.

**P* < 0.05

Table S4. Results of mixed-effects models (*β*, SE, *F*) that compared vegetative phenology and productivity in 2015 with other years from 2003–2017. In addition, the far-right column indicates rank of 2015 versus all 14 other years at survey sites for hoary marmots, in which highest-ranking years had the greatest shift in phenology (earliest start of season, earliest end of season, longest season duration) and highest productivity in North Cascades National Park, Washington, USA.

|  | *β* | SE | *F* | Rank |
| --- | --- | --- | --- | --- |
| Start of season (Julian day) | -23.7* | 2.6 | 80.6 | 1 |
| End of season (Julian day) | -2.04 | 2.61 | 0.61 | 6 |
| Season duration (days) | 21.8* | 3.6 | 37.4 | 1 |
| Cumulative NDVI^a^ | 14.6* | 1.9 | 56.0 | 1 |
| Maximum NDVI | 1.62 | 0.98 | 2.7 | 4 |
| NDVI amplitude | -1.17 | 1.01 | 1.3 | 10 |

^a^Normalized difference vegetation index = NDVI.

**P* < 0.05


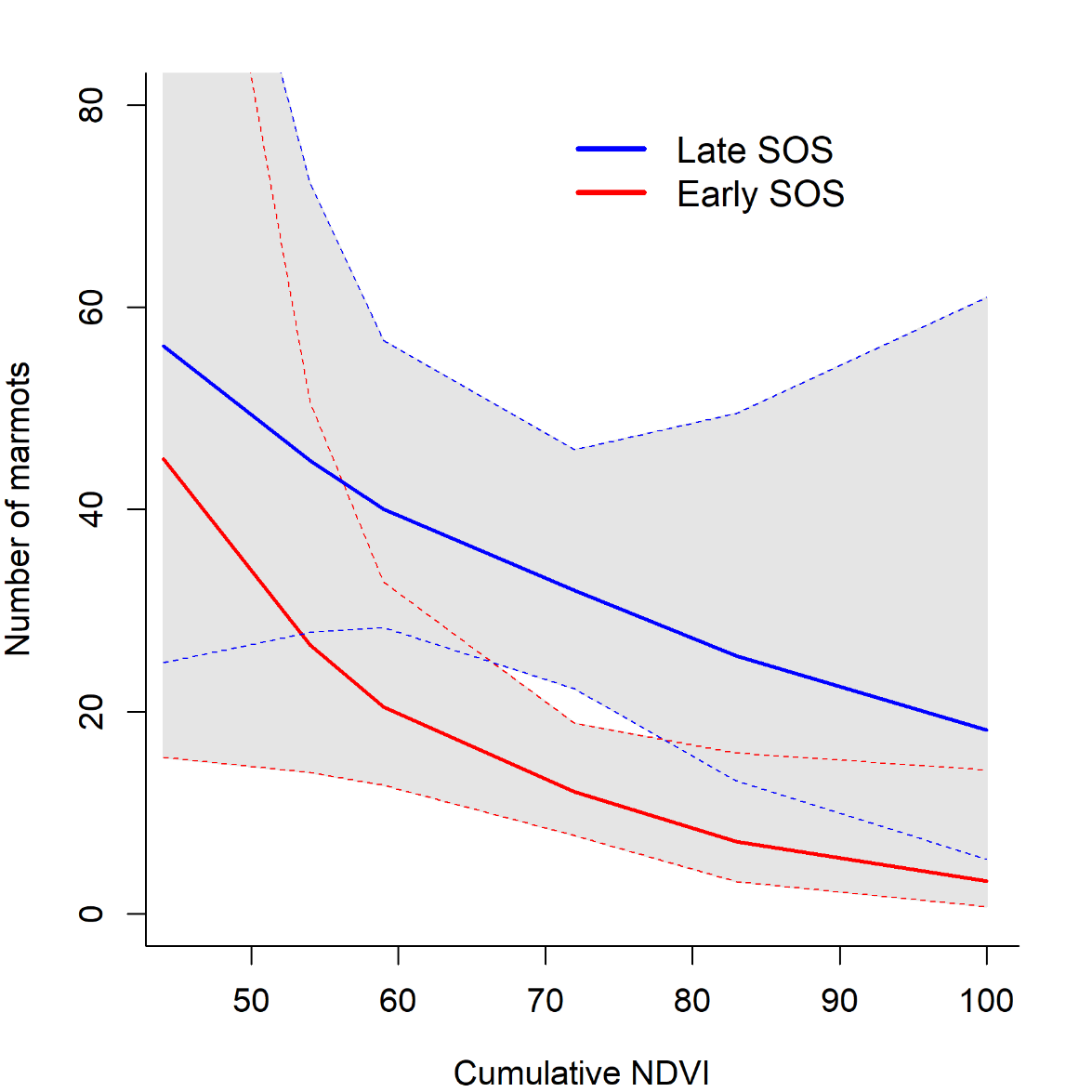


Figure S1. Predicted values (solid lines) and 95% CIs (dashed lines) from the top-ranked model for abundance of hoary marmots, as explained by the interaction of the start of season (SOS) and cumulative normalized difference vegetation index (NDVI) at North Cascades National Park, Washington, USA, 2007-2008, 2016, and 2017. The model was top ranked despite no significant interaction of predictors; an additive model with these predictors was not included in the model set. Each prediction of marmot abundance is calculated with the mean intercept for the random effect of sites. Early and late prediction lines are based on the 10^th^ and 90^th^ percentile values of SOS observed within each dataset. Predictions cover the approximate range of values for the combination of start of season and cumulative NDVI in the datasets.


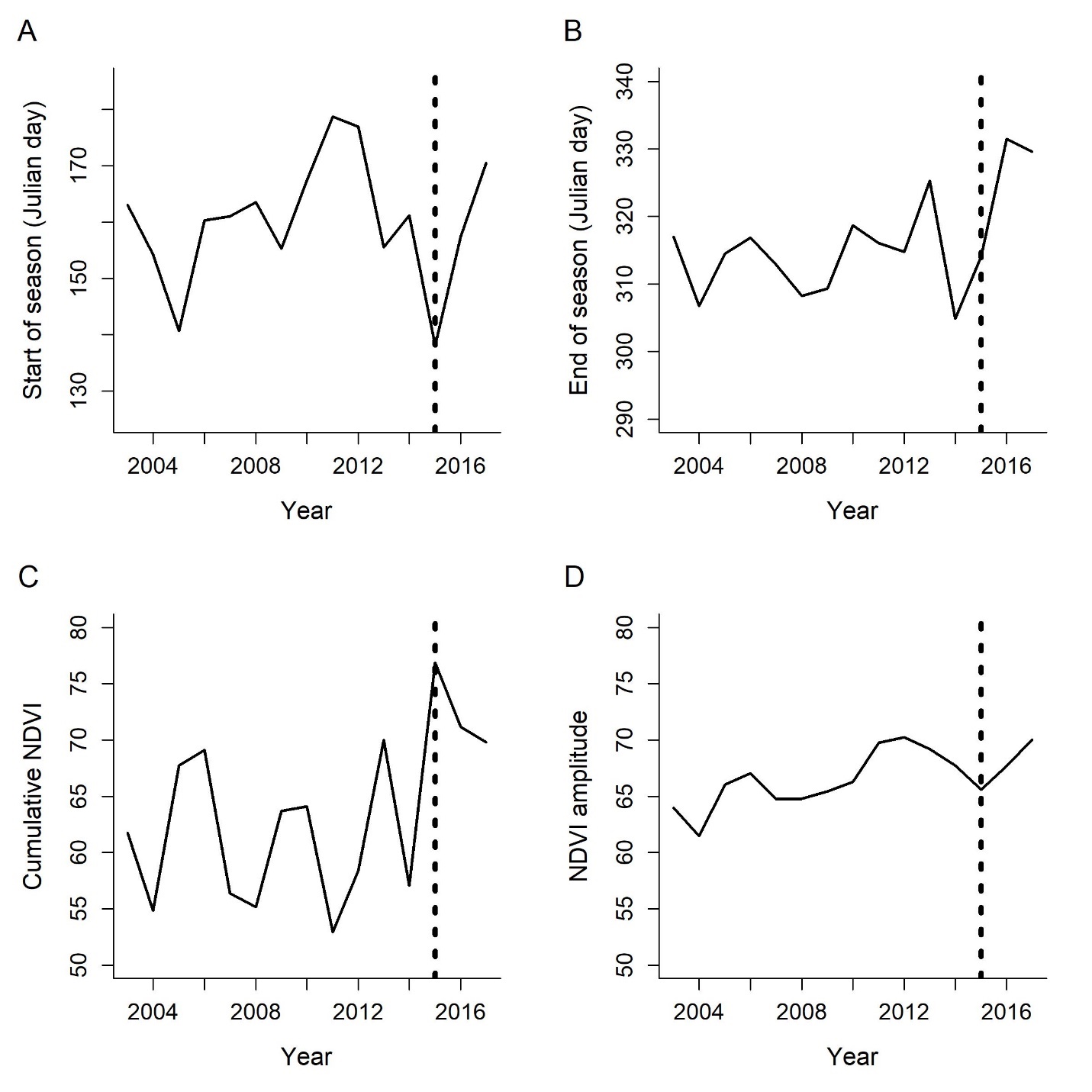


Figure S2. Start of season (A), end of season (B), cumulative normalized difference vegetation index (NDVI) (C), and NDVI amplitude (D) averaged over all sites with surveys for hoary marmots for each year from 2003–2017 in North Cascades National Park, Washington, USA. In each graph, the vertical dashed line marks the record-low snowpack in 2015.

*Literature Cited*

Bates, D., Mächler, M., Bolker, B., & Walker, S. (2015). Fitting linear mixed-effects models using lme4. *Journal of Statistical Software*, 67, 1–48.

Jenkerson, C. B., Maiersperger, T., & Schmidt, G. (2010). eMODIS: A user-friendly data source. Open-File Report 2010–1055, United States Geological Survey, Reston, VA, USA.

Johnston, A. N., Beever, E. A., Merkle, J. A., & Chong, G. (2018). Vegetation responses to sagebrush-reduction treatments measured by satellites. *Ecological Indicators*, 87, 66–76.

Kenward, M. G., & Roger, J. H. (1997). Small sample inference for fixed effects from restricted maximum likelihood. *Biometrics*, 53, 983–997.

Ockendon, N., Baker, D. J., Carr, J. A., White, E. C., Almond, R. E. A, Amano, T., Bertram, E., Bradbury, R. B., Bradley, C., Butchart, S. H. M., Doswald, N., Foden, W., Gill, D. J. C., Green, R. E., Sutherland, W. J., Tanner, E. V. J., & Pearce-Higgins, J. W. (2014). Mechanisms underpinning climatic impacts on natural populations: altered species interactions are more important than direct effects. *Global Change Biology*, 20, 2221–2229.
